# The Role of Aspartate Transaminase to Platelet Ratio Index (APRI) for the Prediction of Non-Alcoholic Fatty Liver Disease (NAFLD) in Severely Obese Children and Adolescents

**DOI:** 10.3390/metabo12020155

**Published:** 2022-02-08

**Authors:** Antonello E. Rigamonti, Adele Bondesan, Eugenia Rondinelli, Silvano G. Cella, Alessandro Sartorio

**Affiliations:** 1 Department of Clinical Sciences and Community Health, University of Milan, 20129 Milan, Italy; silvano.cella@unimi.it; 2 Experimental Laboratory for Auxo-Endocrinological Research, IRCCS, Istituto Auxologico Italiano, 28824 Verbania, Italy; a.bondesan@auxologico.it (A.B.); sartorio@auxologico.it (A.S.); 3 Research Laboratory Unit, IRCCS, Istituto Auxologico Italiano, 28824 Verbania, Italy; e.rondinelli@auxologico.it; 4 Division of Auxology and Metabolic Diseases, IRCCS, Istituto Auxologico Italiano, 28824 Verbania, Italy

**Keywords:** aspartate transaminase to platelet ratio index (APRI), obesity, children/adolescents, biomarker, non-alcoholic fatty liver disease (NAFLD)

## Abstract

The aspartate transaminase to platelet ratio index (APRI) has been proposed as an easy-to-use biochemical marker in obese adults with non-alcoholic fatty liver disease (NAFLD) and non-alcoholic steatotic hepatitis (NASH). The objective of the present study was to evaluate the clinical and predictive value of APRI in a paediatric obese population. Seven hundred fifty-seven obese children and adolescents (BMI standard deviation score, SDS: >2.0; age range: 10–18.5 years), not consuming alcohol and without hepatitis B or C, were recruited after having been screened for NAFLD by ultrasonography. A series of demographic, biochemical and clinical parameters was compared between the two subgroups (with or without NAFLD); the same parameters were correlated with APRI; and finally, univariable and multivariable logistic regression was used to evaluate the predictors of NAFLD. NAFLD was diagnosed in about 39% of the entire paediatric population, predominantly in males and in subjects suffering from metabolic syndrome. APRI was correlated with the waist circumference (WC), high-density lipoprotein cholesterol (HDL-C), uric acid, total bilirubin, C reactive protein (CRP) and systolic blood pressure (SBP). Furthermore, APRI was higher in males than females, but independent from steatosis severity and metabolic syndrome. With the univariable analysis, the BMI SDS, triglycerides (TG), insulin, homeostatic model assessment for insulin resistance (HOMA-IR), APRI, uric acid and metabolic syndrome were positive predictors of NAFLD, with female sex being negative predictor. At multivariable analysis; however, only BMI SDS, TG, HOMA-IR and APRI were positive predictors of NAFLD, with female sex being a negative predictor. The accuracy of APRI as a biochemical marker of NAFLD was about 60%.In conclusion, in a large (Italian) paediatric obese population, parameters, such as BMI SDS, TG, HOMA-IR and APRI, were positive predictors of NAFLD, with female sex being a negative predictor and most of the prediction explained by APRI. Nevertheless, APRI appears to be a simple biochemical marker of liver injury rather than of NAFLD/NASH and, moreover, is endowed with a limited accuracy for the prediction/diagnosis of NAFLD.

## 1. Introduction

The dramatic increase of obesity prevalence worldwide is accompanied by an unsustainable burden of several non-communicable diseases, including non-alcoholic fatty liver disease (NAFLD), which now represents the most frequent cause of chronic liver disease in adults and, not surprisingly, children [1,2]. NAFLD is difficultly summarized in an unique nosographic definition, because it includes a broad spectrum of liver conditions, ranging from fat accumulation in >5% of hepatocytes (non-alcoholic fatty liver) to non-alcoholic steatohepatitis (NASH), in which tissue necro-inflammation is present, hepatocyte injury (the well-known pathological hallmark of hepatocellular ballooning) and, finally, fibrosis at different grading/severity associated with steatosis (non-alcoholic steatofibrosis, NASH) [3].

Reportedly, the natural history of NASH is typically associated with the progression up to cirrhosis and end-stage liver disease, with the need for liver transplantation [4]. By contrast, the natural history of NAFLD/NASH in childhood, including its progression in early adulthood, is not completely known to date, and this deserves further clinical investigation [2]. 

Similarly to adults, in children and adolescents, there is evidence that NAFLD is associated with other obesity-related comorbidities, such as dyslipidaemia, insulin resistance, blood hypertension, hyperuricemia, metabolic syndrome and obstructive sleep apnoea [5,6,7,8,9]. Unfortunately, paediatric NAFLD is typically asymptomatic or presents aspecific symptoms, such as abdominal pain, fatigue, irritability and headache [2]. This explains the difficulty to clinically obtain an early diagnosis [10,11] and to estimate the real prevalence of paediatric NAFLD [1].

The availability of an accurate diagnostic tool is fundamental not only for establishing the diagnosis and disease severity but also for monitoring the disease over time and the effectiveness of new therapeutic interventions. Liver biopsy remains the gold standard method for the diagnosis of NAFLD and for staging disease severity. However, the invasiveness of this procedure represents an important limitation for its repeated use [12].

Apart from imaging techniques, such as ultrasonography, which have the advantage of repeatability, but a non-negligible inter- and intra-operator variability [13], identifying and validating potential novel non-invasive biomarkers, possibly biochemical and easily detectable in serum, is a fascinating area of research in paediatric NAFLD.

Among the different biomarkers proposed to be used for this purpose [2], in recent years, an increasing interest has been addressed regarding the aspartate transaminase to platelet ratio index (APRI), which has been investigated mainly in adult NAFLD and other chronic liver diseases, such as viral hepatitis [14,15,16,17,18]. When compared with liver biopsy and other non-invasive methods, APRI appeared to be a promising biomarker for assessing liver fibrosis/steatosis, with the advantages of being at low cost and repeatable [19]. To our best of knowledge, APRI has been limitedly investigated in paediatric NAFLD.

Thus, based on the previous premises, the aims of the present study, carried out in a large population of obese children and adolescents, echographically screened for NAFDL, were (1) to correlate APRI with a series of demographic, biochemical and clinical parameters and (2) to estimate the predictive value of APRI for the prediction/diagnosis of NAFLD.

## 2. Results

### 2.1. Comparison of Children and Adolescents with and without NAFLD

Table 1 reports the demographic, biochemical and clinical parameters in the two subgroups of subjects with and without NAFLD.

Thirty-nine percent of the 757 children and adolescents (*n* = 295) had NAFLD. Among subjects with NAFLD, 162 (55%) were males and 133 (45%) females, with the sex-related difference being significant. Metabolic syndrome was significantly more prevalent in subjects with than without NAFLD (34% vs. 24%, respectively).

While age was similar in children and adolescents with and without NAFLD, the body mass index standard deviation score (BMI SDS), waist circumference (WC), total cholesterol (T-C), low-density lipoprotein cholesterol (LDL-C), triglycerides (TG), glucose, insulin, homeostatic model assessment for insulin resistance (HOMA-IR), APRI, uric acid, systolic blood pressure (SBP) and diastolic blood pressure (DBP) were significantly higher in children and adolescents with NAFLD than in those without NAFLD, with the exception of high-density lipoprotein cholesterol (HDL-C), which was significantly lower. The total bilirubin and C reactive protein (CRP) did not significantly differ between the two subgroups with and without NAFLD.

### 2.2. Correlations of APRI with Each Demographic, Biochemical and Clinical Parameter

Table 2 reports correlations of APRI with each demographic, biochemical and clinical parameter.

**Figure 1:**
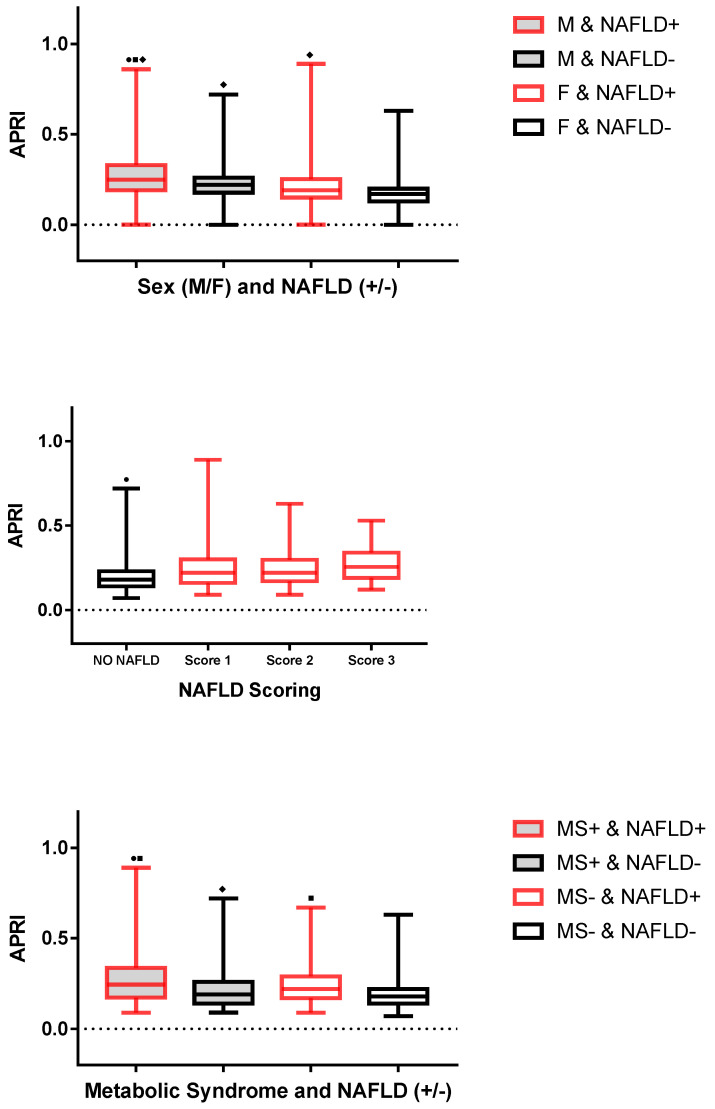
Boxplot of APRI (for specific groups: (**top panel**) sex; (**middle panel**) NAFLD scoring; (**bottom panel**) metabolic syndrome). The segment inside the box is the median (50th percentile); the two segments that constitute the top and bottom of the box are the 25th and 75th percentiles, respectively; and the whiskers are calculated as ±1.5 × IQR. **Top** **Panel**: ■ *p* < 0.05 vs. M and NAFLD−; ● *p* < 0.05 vs. F and NAFLD+; ♦ *p* < 0.05 vs. F and NAFLD−. **Middle** **Panel**: ● *p* < 0.05 vs. score 1 or score 2 or score 3. **Bottom Panel**: ● *p* < 0.05 vs. MS+ and NAFLD−; ■ *p* < 0.05 vs. MS− and NAFLD−; and ♦ *p* < 0.05 vs. MS− and NAFLD+. Abbreviations: F, female; NAFLD, non-alcoholic fatty liver disease; M, male; and MS, metabolic syndrome.

APRI was positively correlated with the WC, uric acid, total bilirubin and SBP, while it was negatively correlated with HDL-C and CRP. No significant correlations of APRI were detected with age, BMI SDS, T-C, LDL-C, TG, glucose, insulin, HOMA-IR and DBP. Figure 1 graphically summarizes the statistical analysis of APRI in children and adolescents with and without NAFLD, divided into specific subgroups: sex (top panel), steatosis scoring (middle panel) and metabolic syndrome (bottom panel).

Males with NAFLD had significantly higher APRI than did males without NAFLD and females independently from the diagnosis of NAFLD. While there was no significant difference in APRI among males without NAFLD and females with NAFLD, males without NAFLD had significantly higher APRI than did females without NAFLD. APRI was significantly higher in females with NAFLD than in those without NAFLD.

APRI was significantly lower in the subgroup without NAFLD than in those with NAFLD at different scores of steatosis (i.e., 1, 2 and 3). No significant differences in APRI were found when comparing the different steatosis scores (i.e., 1 vs. 2, 2 vs. 3 and 1 vs. 3). APRI was significantly higher in children and adolescents with NAFLD than in those without NAFLD, independently from the diagnosis of metabolic syndrome.

### 2.3. Univariable Analysis of NAFLD Predictors

Table 3 summarizes the results of the univariable analysis of NAFLD predictors. Confirming the results of between-group comparisons (Table 1), sex, BMI SDS, TG, insulin, HOMA-IR, APRI, uric acid and metabolic syndrome were significantly associated with NAFLD, while age, WC, T-C, HDL-C, LDL-C, glucose total bilirubin, DBP and SBP were not.

In particular, NAFLD was 0.38-times more likely in females and 1.63-times less likely in children and adolescents without metabolic syndrome. An increase of 1 SDS of BMI was associated with a doubling in the odds of NAFLD. An increase of 10 mg/dL of TG or 10 µIU/L of insulin levels increased the odds of NAFLD of about 100%. An increase of 1 of HOMA-IR, 1 mg/dL of uric acid or 1 of APRI was associated with a 100%, 135% or 250%, respectively, increase in the odds of NAFLD. 

### 2.4. Multivariable Analysis of NAFLD Predictors

Adding the most relevant significant predictors of NAFLD at univariable analysis (Table 3) in a multivariable logistic regression model, sex, BMI SDS, TG, HOMA-IR and APRI were identified as significant predictors (Table 4). In particular, as determined by standardized regression coefficients, APRI was the strongest multivariable predictor of NAFLD. In comparison, female sex was responsible for 16% of the variability explained by APRI, BMI SDS for 10%, HOMA-IR for 8% and TG for 0.2%. 

### 2.5. Accuracy of APRI as a Marker of NAFLD

**Figure 2:**
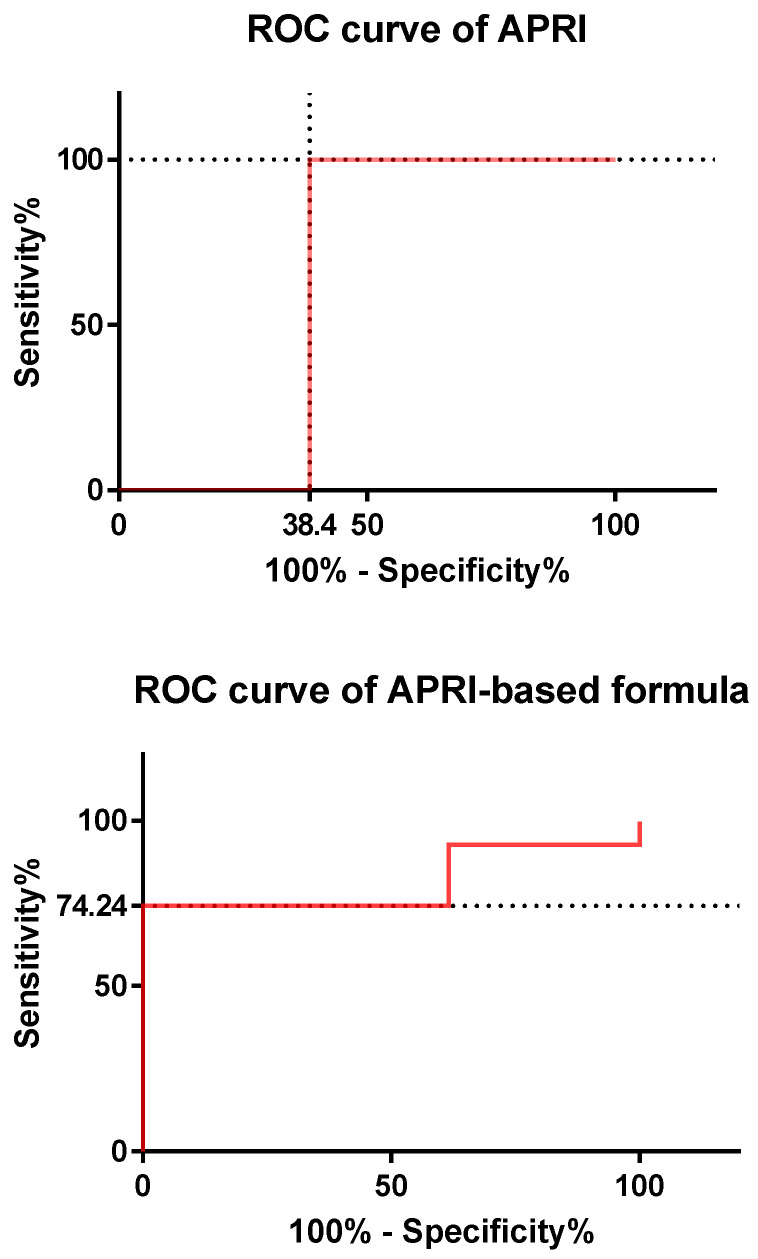
ROC (receiver operating characteristic) curves for APRI (**top panel**) and the multivariable logistic regression model (**bottom panel**). The dotted lines indicate the values of sensitivity/specificity at the corresponding cut-off. See the text for further details. Abbreviation: ROC, receiver operating characteristic.

The use of APRI as continuous variable (Figure 2) permitted the prediction of NAFLD with an accuracy of 61.6% (*p* < 0.0001) with a sensitivity of 100% and specificity of 61.6% at a cut-off of APRI > 0.03571.

On the contrary, the use of the formula deriving from the multivariable logistic regression model [Logit P = −3.815 − (0.681 × female sex) + (0.416 × BMI SDS) + (4.232 × APRI) − (0.0443 × insulin) + (0.346 × HOMA IR) − (0.172 × metabolic syndrome) + (0.00709 × triglycerides) + (0.0843 × uric acid)] (Figure 2) improved the accuracy to 81.34% (*p* < 0.0001) with a sensitivity of 74.24% and specificity of 100% at a cut-off of Logit P < −0.00146.

## 3. Discussion

The present clinical study, carried out in a large Italian paediatric obese population characterized by a 39% prevalence of echographically diagnosed NAFLD, confirmed the notion of a higher prevalence of NAFLD in obese males compared with in females. Furthermore, our results show that female sex was a negative predictor of NAFLD, and APRI was higher in male over female obese children and adolescents with NAFLD.

Though our paediatric population included subjects at different pubertal stages (which, as demonstrated in a previous work, have different distributions in NAFLD [20]), oestrogens are likely to be implicated in this anti-steatotic effect in females, as these sex steroid hormones are endowed with anti-inflammatory, insulin-sensitizing and cytoprotective effects [21]. Furthermore, oestrogens are known to promote a gynecoid fat distribution, impeding abdominal (visceral) fat accumulation, which exerts a pathogenetic role in metabolic syndrome [22]. 

In the present study, metabolic syndrome was more prevalent in obese children and adolescents with than without NAFLD—a not surprising finding because the diagnostic criteria for metabolic syndrome encompass cardiometabolic risk factors for NAFLD: predominantly visceral obesity, hypertriglyceridemia and insulin resistance [23,24]. 

Differently from the univariable analysis, in our multivariable analysis of NAFLD predictors, metabolic syndrome was not a NAFLD predictor, being instead NAFLD predictors other parameters such as BMI SDS, TG and HOMA-IR, which can be considered “surrogates” of the (“true”) IDF diagnostic criteria of metabolic syndrome (for WC, dyslipidemia and diabetes mellitus, respectively) [23]. 

Similarly, APRI was not associated with metabolic syndrome, and the difference in APRI between the NAFLD groups with and without metabolic syndrome was not significant. On the contrary, APRI was significantly correlated with WC and SBP (positively) or HDL-C (negatively), which are, indeed, diagnostic criteria of metabolic syndrome [23].

Based on previous considerations, the relationship of NAFLD or APRI with metabolic syndrome or the single diagnostic criteria of metabolic syndrome may appear puzzling but does denote the pathogenic importance of specific cardiometabolic risk factors (e.g., visceral obesity, hypertriglyceridemia and insulin resistance) rather than an omnicomprehensive but not universally accepted nosographic definition of metabolic syndrome [25].

Due to the coexistence of visceral obesity, insulin resistance and dyslipidemia, NAFLD is considered to be the hepatic manifestation of metabolic syndrome [26]. In recent years, these relationships between NAFLD and cardiometabolic risk factors (or metabolic syndrome) in adults led to a new term combining both of these conditions, called metabolic dysfunction-associated fatty liver disease (MAFLD). Interestingly, based on these findings, some authors proposed a set of criteria that might be useful to diagnose MAFLD in children and adolescents [27].

In the present study, despite an evident sex-related dependence, APRI was not associated with steatosis severity, evaluated by means of a somewhat raw echographic scoring. Furthermore, APRI was negatively correlated with CRP—a not surprising finding because systemic inflammation increases both acute phase plasma proteins and the platelet count, which appears as the denominator in the formula of APRI [28].

Therefore, based on the results of the correlation analysis, APRI is likely to be a scarcely specific biochemical marker of liver injury rather than of NAFLD/NASH, in which fibrosis is only one of the histopathological component [29]. Further clinical studies are needed to combine histopathological evaluation (i.e., liver biopsy, an invasive method for children and adolescents) with biochemical measurements (such as APRI). The “limited” clinical value of APRI is also reflected by the “limited” predictive accuracy of APRI when used as a single marker for the prediction/diagnosis of NAFLD (about 60%, not greatly different from the range reported in medical literature for adult NAFLD, i.e., 66–74%) [30]. 

As reported in the present study, diagnostic performance could be improved only by using the complex formula deriving from multivariable analysis (with an accuracy of about 80%), in which APRI explained the most variability of the model, with a negligible role for the other predictors: female sex, BMI SDS, TG and HOMA-IR. In our opinion, in comparison to APRI as single or composite marker, new more promising non-invasive markers for NAFLD/NASH are currently under investigation [30].

Before closing, some limitations of our clinical study should be mentioned. First, our clinical study was performed in an “Italian” paediatric obese population, and thus differences from the results obtained in other clinical studies [31,32,33] might depend on environmental, sociocultural, behavioural and epigenetic/genetic factors that are specific to the Italian context. 

Second, the diagnosis of NAFLD was made by using an ultrasonographic technique, which, as known, underestimates the number and severity of steatosis cases with a wide inter- and intra-operator variability [13]. This issue is of difficult experimental solution, due to the invasiveness of liver biopsy and to the high-cost of other imaging techniques, such as magnetic nuclear resonance [2]. However, starting in the last decade, new ultrasound-based techniques to estimate the stage of liver fibrosis are becoming widely available. Particularly, multiparametric ultrasound-based tools that are able to quantify both steatosis and fibrosis are starting to be used also in clinical practice [34,35].

Third, the conclusions of the present study should be cautiously extrapolated to different contexts because a control group consisting of normal-weighted children and adolescents with NAFLD was missing. Additionally, there is the need to validate our NAFLD predictors in a “real-world” context, which, herein, were only statistically derived. This issue might be the rationale for a future clinical study.

## 4. Materials and Methods

### 4.1. Subjects

Seven hundred fifty-seven obese children and adolescents were consecutively recruited at the Division of Auxology, Istituto Auxologico Italiano (Piancavallo, Verbania, Italy), where they were hospitalized for a 3-week period of in-hospital multidisciplinary metabolic rehabilitation. Inclusion criteria were: (1) age > 10 and <19 years; (2) BMI SDS ≥ 2.0 for sex and age using the Italian reference curves [36]; (3) essential obesity; (4) absence of any concomitant drug treatment; (5) abstinence from alcohol; and (6) absence of positive serological markers of hepatitis B virus (HBV) and hepatitis C virus (HCV).

Alcohol consumption was determined by interview with the children/adolescents and/or their parents. HBV surface antigen (HBsAg) and antibodies against HCV were measured to rule out hepatitis B and C. 

### 4.2. Diagnosis and Scoring of NAFLD

Liver ultrasonography was performed by the same operator implementing standard criteria [37,38]. Mild steatosis (score 1) was defined as slightly increased liver echogenicity with normal vessels and absent posterior attenuation, moderate steatosis (score 2) as moderately increased liver echogenicity with partial dimming of vessels and early posterior attenuation and severe steatosis (score 3) as diffusely increased liver echogenicity with absence of visible vessels and heavy posterior attenuation. NAFLD was operationally defined as any degree of fatty liver in the absence of HBV and HCV infection and alcohol intake. A normal liver was defined as the absence of fatty liver. 

### 4.3. Anthropometric Measurements

A scale with a stadiometer was used to determine height and weight (Wunder Sa.Bi., WU150, Trezzo sull’Adda, Italy). WC was measured with a flexible tape in standing position, halfway between the inferior margin of the ribs and the superior border of the crista. BMI was calculated as weight (kg) to squared stature (m); BMI SDS was calculated from Italian reference data using the LMS method [36]. 

### 4.4. Metabolic Variables

Blood samples (about 10 mL) were collected at around 8:00 AM after an overnight fast. Aspartate aminotransferase (AST), T-C, HDL-C, LDL-C, TG, total bilirubin, uric acid, glucose, insulin and CRP were measured. Colorimetric enzymatic-assays (Roche Diagnostics, Monza, Italy) were used to determine serum AST, T-C, LDL-C, HDL-C, TG, uric acid and total bilirubin levels. The sensitivities of the method for each parameter were 5 U/L, 3.86 mg/dL, 3.87 mg/dL, 3.09 mg/dL, 8.85 mg/dL, 0.2 mg/dL and 0.146 mg/dL, respectively.

The serum glucose level was measured by the glucose oxidase enzymatic method (Roche Diagnostics, Monza, Italy). The sensitivity of the method was 2 mg/dL. The serum insulin concentration was determined by a chemiluminescent immunometric assay, using a commercial kit (Elecsys Insulin, Roche Diagnostics, Monza, Italy). The sensitivity of the method was 0.2 µIU/mL.

Insulin resistance was estimated using the HOMA-IR method [39]. CRP was measured using an immunoturbidimetric assay (CRP RX, Roche Diagnostics GmbH, Mannheim, Germany). The sensitivity of the method was 0.03 mg/dL. APRI was calculated by means of the following formula: (AST [IU/L]/40)/platelet count [10^9^/L] * 100.

### 4.5. Evaluation of Blood Pressure

Blood pressure was measured on the right arm, using a sphygmomanometer with appropriate paediatric cuff size, with the subject in a seated position and relaxed condition. The procedure was repeated three times at 10 min intervals; the means of the three values for SBP and DBP were recorded.

### 4.6. Definition of Metabolic Syndrome

According to the IDF (International Diabetes Federation) criteria for diagnosis of metabolic syndrome in children and adolescents [23], our patients were considered positive for the presence of metabolic syndrome if they had abdominal obesity (WC ≥ 90th percentile [40] for ages < 16 years, and ≥94 cm for males and ≥80 cm for female for ages > 16 years) plus two or more of the following factors: 

(i) increased TG level: ≥150 mg/dL (1.7 mmol/L) for ages < 16 years and the same cut-off or specific treatment for this lipid abnormality for ages > 16 years;

(ii) reduced HDL-C: <40 mg/dL (1.03 mmol/L) for males and females for ages < 16 years, and <40 mg/dL for males and <50 mg/dL (1.29 mmol/L) for females, or specific treatment for this lipid abnormality for ages > 16 years;

(iii) increased BP: SBP ≥ 130 mmHg or DBP ≥ 85 mmHg for ages < 16 years, and same cut-off or treatment of previously diagnosed hypertension for ages > 16 years; and

(iv) increased fasting glucose concentration ≥ 100 mg/dL (5.6 mmol/L) or previously diagnosed type 2 diabetes mellitus for all ages.

### 4.7. Statistical Analysis

The Sigma Stat 3.5 statistical software package (Systat Software, San Jose, CA, USA) was used for data analyses and GraphPad Prisma 7.0 software (GraphPad Software, San Diego, CA, USA) for data plotting. Values of continuous variables were expressed as medians and interquartile ranges (IQR) because of the failure of normalcy. Between-group comparisons of continuous variables were performed with the rank sum test and those of ordinal variables with chi-square test. 

A one-way analysis of variance (ANOVA) on ranks, followed by the Tukey’s test, was applied when there was the need of multiple comparisons. Correlations of APRI with each demographic, biochemical and clinical parameter were determined by using Spearman’s rank correlation coefficient. Logistic regression was used to evaluate the association between potential predictors and NAFLD, coded as present vs. absent. Apart from sex and metabolic syndrome, all predictors were evaluated as continuous variables. Age, WC, T-C, HDL-C, LDL-C, TG, glucose, insulin, and systolic and diastolic blood pressures were divided by 10 before use in the logistic regression models. 

The most relevant significant predictors of NAFLD at univariable analysis were evaluated in a multivariable logistic regression model. Raw data of APRI and probabilities obtained by multivariable logistic regression were used to draw ROC (receiver operating characteristic) curves using the DeLong, DeLong and Clarke–Pearson method, and the area under the ROC curves (AUC) was used to assess the accuracy of APRI and the logistic regression model. Statistical significance was set to a value of *p* < 0.05 for all tests. 

## 5. Conclusions

In a large paediatric obese population, characterized by a 39% prevalence of echographic NAFLD diagnosis, we found that BMI SDS, TG, HOMA-IR and APRI, but not metabolic syndrome and uric acid, were positive predictors of NAFLD, with female sex being a negative predictor. APRI appears to be a simple biochemical marker of liver injury rather than of NAFLD/NASH and, moreover, is endowed with a limited accuracy for the prediction/diagnosis of NAFLD. 

New biochemical markers that are simple-to-measure, easy-to-use and at low-cost, such as APRI, should be identified in future clinical studies. This is an urgent need due to the dramatic prevalence of NAFLD in paediatric obesity worldwide and to the rapid monitoring of the effectiveness of our anti-obesity interventions.

## Figures and Tables

**Table 1:** Demographic, clinical and biochemical parameters in the two subgroups of obese paediatric population without NAFLD (NAFLD −) and with NAFLD (NAFLD +).

Parameter	NAFLD −	NAFLD +	*p*
Sex (M/F)	141/321	162/133	<0.001
Age (years)	15.0 (13.4–16.5) [10.0–18.5]	15.0 (12.5–16.6) [10.0–18.3]	0.297
BMI-SDS	2.9 (2.5–3.2) [2.0–4.5]	3.1 (2.7–3.5) [2.0–4.6]	<0.001
WC (cm)	111.0 (103.0–122.0) [69.0–160.0]	121.0 (113.0–131.0) [93.0–166.0]	<0.001
T-C (mg/dL)	157.5 (141.0–180.2) [100.0–425.0]	165.0 (143.0–186.0) [54.0–291.0]	0.028
HDL-C (mg/dL)	42.0 (36.0–49.2) [17.0–118.0]	40.0 (35.0–47.0) [19.0–77.0]	0.005
LDL-C (mg/dL)	99.0 (83.0–118.0) [45.0–357.0]	108.0 (87.0–126.0) [18.0–218.0]	0.004
Triglycerides (mg/dL)	83.0 (65.0–110.2) [15.0–340.0]	96.0 (77.0–126.0) [14.0–293.0]	<0.001
Glucose (mg/dL)	80.0 (77.0–85.0) [64.0–130.0]	82.0 (78.0–86.0) [69.0–108.0]	0.007
Insulin (µIU/L)	11.7 (7.8–16.8) [2.0–75.4]	16.4 (10.9–22.0) [2.0–47.5]	<0.001
HOMA-IR	2.3 (1.5–3.3) [0.3–14.6]	3.2 (2.2–4.5) [0.3–12.6]	<0.001
APRI	0.1 (0.1–0.2) [0.0–0.7]	0.2 (0.1–0.3) [0.0–0.8]	0.036
Uric acid (mg/dL)	5.8 (5.1–6.7) [0.7–10.5]	6.6 (5.5–7.5) [0.2–10.1]	<0.001
Total bilirubin (mg/dL)	0.5 (0.4–0.7) [0.2–2.2]	0.5 (0.4–0.7) [0.1–2.5]	0.310
CRP (mg/dL)	0.3 (0.2–0.6) [0.0–8.7]	0.4 (0.2–0.7) [0.0–4.0]	0.095
SBP (mmHg)	120.0 (120.0–130.0) [100.0–170.0]	130.0 (120.0–130.0) [90.0–180.0]	<0.001
DBP (mmHg)	80.0 (70.0–80.0) [50.0–110.0]	80.0 (80.0–80.0) [60.0–110.0]	<0.001
Metabolic syndrome (yes/no)	111/351	100/195	0.004

Note: data are expressed as medians, 25–75% interquartile ranges (round brackets) and minimum and maximum values (square brackets); statistical analysis was performed with chi-square test and rank sum test. Abbreviations: APRI, AST (i.e., aspartate aminotransferase) to platelet count; BMI, body mass index; CRP, C-reactive protein; DBP, diastolic blood pressure; F, female; HDL-C, high-density lipoprotein cholesterol; HOMA-IR, homeostasis model assessment insulin resistance; LDL-C, low-density lipoprotein cholesterol; M, male; SDS, standard deviation score; T-C, total cholesterol; and WC, waist circumference.

**Table 2:** Correlations of APRI with the other demographic, clinical and biochemical parameters.

Parameter	r (95% CI)	*p*
Age (years)	0.010 (−0.063–0.083)	0.7768
BMI SDS	0.032 (−0.040–0.106)	0.3658
WC (cm)	0.112 (−0.122–0.023)	0.0020
T-C (mg/dL)	−0.021 (−0.095–0.051)	0.5483
HDL-C (mg/dL)	−0.115 (−0.187–−0.042))	0.0015
LDL-C (mg/dL)	0.000 (−0.073–0.073)	0.9970
Triglycerides (mg/dL)	0.061 (−0.011–0.134)	0.0894
Glucose (mg/dL)	0.005 (−0.067–0.078)	0.8763
Insulin (µIU/L)	0.028 (−0.045–0.101)	0.4400
HOMA-IR	0.023 (−0.850–0.096)	0.5224
Uric acid (mg/dL)	0.229 (0.158–0.297)	<0.0001
Total bilirubin (mg/dL)	0.138 (0.065–0.209)	0.0001
CRP (mg/dL)	−0.154 (−0.225–−0.082)	<0.0001
SBP (mmHg)	0.085 (0.011–0.157)	0.0193
DBP (mmHg)	0.059 (−0.013–0.132)	0.1016

Abbreviations: 95% CI, 95% confidence interval; for the other abbreviations see the legend of Table 1.

**Table 3:** Univariable analysis of predictors for NAFLD.

Predictor	OR (95% CI)	*p*
Female	0.367 (0.270–0.497)	<0.001
Age (years/10)	1.000 (0.978–1.022)	0.984
BMI SDS	2.164 (1.637–2.862)	<0.001
WC (cm/10)	1.002 (0.999–1.004)	0.284
T-C (mg/dL/10)	1.000 (0.998–1.002)	0.864
HDL-C (mg/dL/10)	0.997 (0.990–1.004)	0.418
LDL-C (mg/dL/10)	1.000 (0.998–1.003)	0.763
Triglycerides (mg/dL/10)	1.003 (1.001–1.006)	0.018
Glucose (mg/dL/10)	1.000 (0.996–1.004)	0.869
Insulin (µIU/L/10)	1.027 (1.011–1.044)	0.001
HOMA-IR	1.351 (1.231–1.482)	<0.001
APRI	250.510 (47.891–1310.384)	<0.001
Uric acid (mg/dL)	1.438 (1.277–1.621)	<0.001
Total bilirubin (mg/dL)	0.833 (0.506–1.372)	0.473
CRP (mg/dL)	0.958 (0.779–1.179)	0.688
SBP (mmHg/10)	1.001 (0.998–1.003)	0.593
DBP (mmHg/10)	1.001 (0.997–1.005)	0.658
Metabolic syndrome	1.631 (1.180–2.255)	0.003

Note: the values of *p* refer to the likelihood ratio test; abbreviations: OR, odds ratio; for the other abbreviations see the legend of Table 1.

**Table 4:** Multivariable analysis of predictors for NAFLD.

Predictor	OR (95% CI)	*p*	Coefficient
Female	0.506 (0.353–0.726)	<0.001	−0.681
BMI SDS	1.516 (1.081–2.126)	0.016	0.416
Triglycerides (mg/dL/10)	1.007 (1.00–1.012)	0.008	0.007
HOMA-IR	1.413 (1.226–1.629)	<0.001	0.346
APRI	68.853 (12.343–384.091)	<0.001	4.232
Uric acid (mg/dL)	1.088 (0.947–1.250)	0.234	0.084
Metabolic syndrome	0.842 (0.571–1.242)	0.386	−0.172

Note: the values of *p* refer to the Wald test, while coefficient represents the standardized regression coefficient; for the other abbreviations see the legend of Table 1.

## Data Availability

The datasets used and/or analysed in the present study are available from the corresponding author upon a reasonable request. The data are not publicly available due to ethical restrictions.

## Abbreviations

AUC	area under curve
BMI	body mass index
CI	confidence interval
CRP	C-reactive protein
DBP	diastolic blood pressure
F	female
HDL-C	high-density lipoprotein cholesterol
HOMA-IR	homeostatic model assessment for insulin resistance
IDF	International Diabetes Federation
IQR	interquartile range
LDL-C	low-density lipoprotein cholesterol
NAFLD	non-alcoholic fatty liver disease
NASH	non-alcoholic steatotic hepatitis
M	male
OR	odd ratio
ROC	receiver operating characteristic
SBP	systolic blood pressure
SD	standard deviation
SDS	standard deviation score
T-C	total cholesterol
TG	triglycerides
WC	waist circumference.
