# Correction: Rigamonti et al. The Role of Aspartate Transaminase to Platelet Ratio Index (APRI) for the Prediction of Non-Alcoholic Fatty Liver Disease (NAFLD) in Severely Obese Children and Adolescents. *Metabolites* 2022, *12*, 155

**DOI:** 10.3390/metabo12060555

**Published:** 2022-06-17

**Authors:** Antonello E. Rigamonti, Adele Bondesan, Eugenia Rondinelli, Silvano G. Cella, Alessandro Sartorio

**Affiliations:** 1 Department of Clinical Sciences and Community Health, University of Milan, 20129 Milan, Italy; silvano.cella@unimi.it; 2 Experimental Laboratory for Auxo-Endocrinological Research, IRCCS, Istituto Auxologico Italiano, 28824 Verbania, Italy; a.bondesan@auxologico.it (A.B.); sartorio@auxologico.it (A.S.); 3 Research Laboratory Unit, IRCCS, Istituto Auxologico Italiano, 28824 Verbania, Italy; e.rondinelli@auxologico.it; 4 Division of Auxology and Metabolic Diseases, IRCCS, Istituto Auxologico Italiano, 28824 Verbania, Italy

## Missing Funding

In the original publication [1], the Italian Ministry of Health (funder to Istituto Auxologico Italiano, IRCCS, Milan, Italy) was not included in the funding section. The authors apologize for any inconvenience caused and state that the scientific conclusions are unaffected. This correction was approved by the Academic Editor. The original publication has also been updated.
